# Chronic graft-versus-host disease myelitis successfully treated with rituximab

**DOI:** 10.1007/s12185-025-03936-y

**Published:** 2025-01-31

**Authors:** Emi Yokoyama, Yuta Hasegawa, Kentaro Wakaki, Touma Suzuki, Sayaka Kajikawa, Minoru Kanaya, Koh Izumiyama, Makoto Saito, Masanobu Morioka, Jun Nagai, Tomoe Ichiki, Ryo Kikuchi, Satomi Okada, Hiroyuki Ohigashi, Hideki Goto, Masahiro Onozawa, Daigo Hashimoto, Akio Mori, Takanori Teshima, Takeshi Kondo

**Affiliations:** 1 Blood Disorders Center, Aiiku Hospital, S4W25, Chuo-ku, Sapporo, 064-0804 Japan; 2 Department of Hematology, Hokakido University Hospital, Sapporo, Japan; 3 Department of Rehabilitation, Aiiku Hospital, Sapporo, Japan

**Keywords:** Allogeneic stem-cell transplantation, Atypical chronic graft-versus-host disease, Central nervous system, Myelitis, Rituximab

## Abstract

Chronic graft-versus-host disease (cGVHD) is a major serious complication after allogeneic stem-cell transplantation (allo-HSCT), and often mimics autoimmune diseases. Central nervous system (CNS) symptoms are rare manifestations of cGVHD, and are difficult to diagnose. CNS manifestations of cGVHD were discussed in the 2020 National Institutes of Health cGVHD Consensus Project as one of the “atypical cGVHD manifestations” with involvement of various organ systems other than classical cGVHD organs. We experienced a case of myelitis after allo-HSCT diagnosed as cGVHD of the CNS. The neurological symptoms progressed after corticosteroid pulse therapy, resulting in severe paralysis and paresthesia of the lower extremities. The clinical course and magnetic resonance imaging findings showed some similarities with multiple sclerosis. We decided to use rituximab after the patient became refractory to corticosteroids because rituximab has been reported to be effective in multiple sclerosis by suppressing *B* cells on both sides of the blood–brain barrier. Rituximab was effective for the neurologic symptoms in our case. In atypical cGVHD, treatments used in corresponding autoimmune diseases may be reasonable and effective.

## Introduction

Chronic graft-versus-host disease (cGVHD) is a major serious complication after allogeneic hematopoietic stem-cell transplantation (allo-HSCT). It can affect multiple tissues and organs, resulting in pathophysiological conditions mimicking autoimmune diseases. Central nervous system (CNS) manifestations of cGVHD rarely occur but have been sporadically recognized in the clinical setting. Historically, myositis and polymyositis were the only distinctive neuromuscular manifestations of cGVHD, and CNS manifestations were less clearly associated with cGVHD [1]. Openshaw proposed criteria for diagnosis of cGVHD of the CNS in 2009 [2] (Table 1). Co-occurrence of cGVHD manifestations in other organs, such as the mouth, skin, eye, gastrointestinal tract, liver, lung, or joint, and ruling out of other CNS diseases are mandatory criteria. Corresponding MRI abnormality, abnormal results of cerebrospinal fluid studies, pathologic findings with brain biopsy or post-partum examination, and response to immunosuppressive therapy are facultative criteria. Definite diagnosis can be made when all six criteria are fulfilled. For possible diagnosis, all mandatory criteria and at least two facultative criteria must be met.

**Table 1:** Proposed criteria of CNS manifestations of cGVHD

Mandatory criteria	
1	Occurrence with cGVHD affecting other organs
2	Neurologic signs of the CNS involvement without other explanation

Facultative criteria	
3	Corresponding brain MRI abnormality
4	Abnormal CSF studies (pleocytosis, elevated protein or immunoglobulin G, oligoclonal bands)
5	Pathologic brain biopsy or post-mortem examination
6	Response to immunosuppressive therapy

Source: Adapted from Openshaw [2]

“Definite” diagnosis: All six criteria must be met

“Possible” diagnosis: All mandatory criteria and at least two facultative criteria must be met

The 2005, 2014, and 2020 National Institutes of Health (NIH) consensus projects have defined diagnostic and distinctive criteria for 8 target cGVHD organs: skin, mouth, eyes, lungs, musculoskeletal system, gastrointestinal tract, genitourinary tract, and liver [1, 3, 4]. These are so-called classical cGVHD organs. As part of the 2020 NIH cGVHD Consensus Project, “atypical cGVHD”, which means various manifestations of involvement of organ systems other than those in classical cGVHD, was discussed [5]. Atypical cGVHD includes the hematopoietic system, endothelium, musculoskeletal system, central and peripheral nervous systems, kidneys, and serous membranes. CNS manifestations are also recognized in acute GVHD phase as one of nonclassical acute GVHD and have been attracting attention [6]. cGVHD of the CNS is very rare, and the lack of understanding of its pathology and treatment strategy often leads to a dismal prognosis.

We experienced a case of myelitis after allo-HSCT that was refractory to repeated corticosteroid pulse therapy and was successfully treated with rituximab.

## Case report

A 40-year-old male was diagnosed as having myelodysplastic syndrome with multilineage dysplasia (MDS-MLD) with intermediate risk score of revised international prognostic scoring system. He was considered eligible for allo-HSCT because of high blood transfusion dependency. He received bone marrow transplantation from an HLA-DR 1-locus mismatched unrelated allogeneic male donor. The pre-transplant procedure consisted of administration of fludarabine (30/m^2^, day-7 to day-2), intravenous busulfan (3.2 mg/kg, day-7 to day-4), and melphalan (40 mg/m^2^, day-3 to day-2). Tacrolimus, short-term methotrexate (10-7-7 mg/m^2^) and anti-thymocyte globulin (1 mg/kg, day-2) were used for GVHD prophylaxis. Neutrophil engraftment was achieved on day + 14. There was no obvious GVHD during the acute phase, and he was discharged on day + 35 without any other complications. Stage 3 skin GVHD occurred during tapering of tacrolimus on day + 89 after allo-HSCT. The skin manifestation ameliorated after administration of prednisolone at a dose of 0.5 mg/kg. Prednisolone was tapered and maintained at 10 mg/day.

**Fig. 1:**
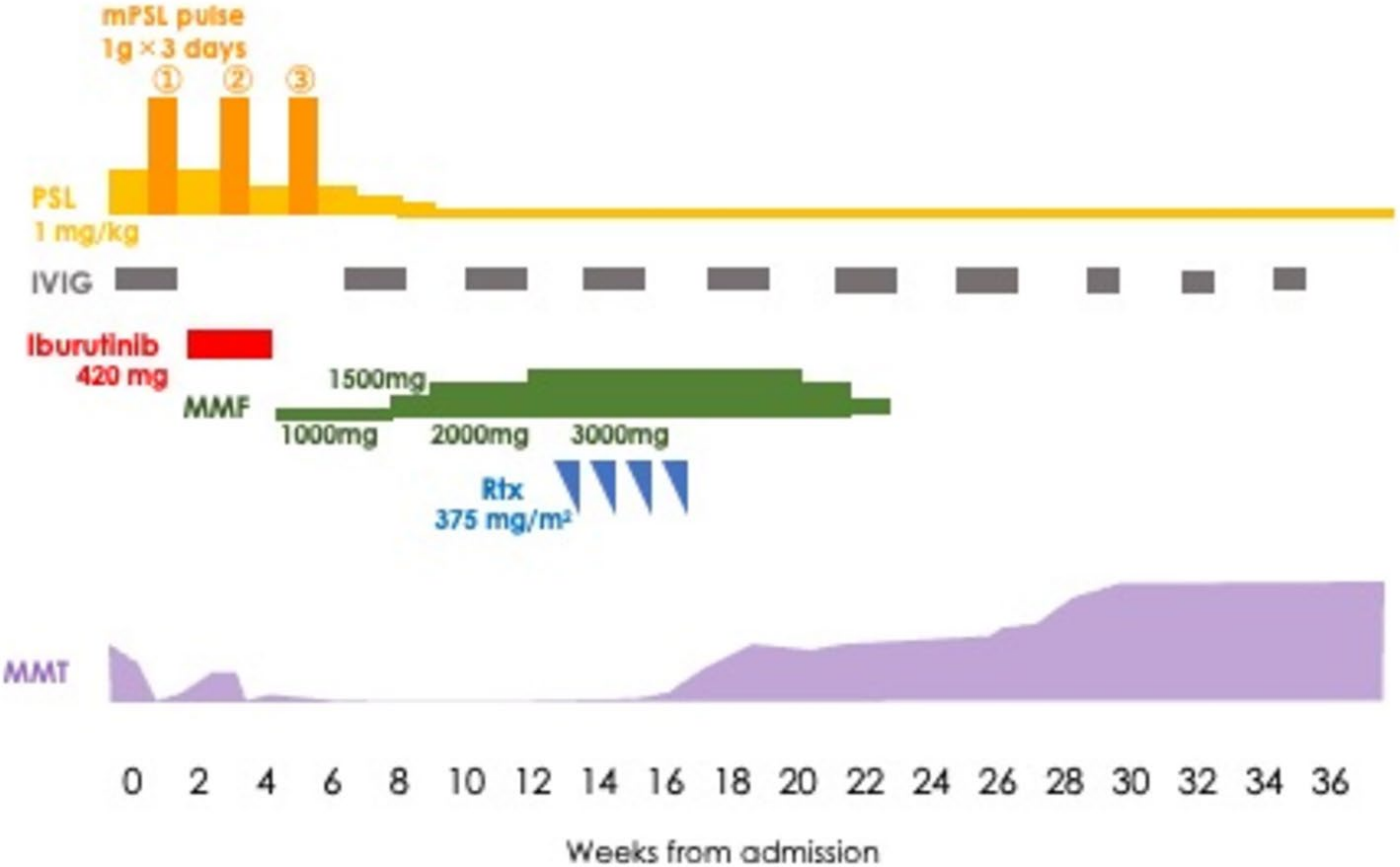
Clinical course of the case

**Fig. 2:**
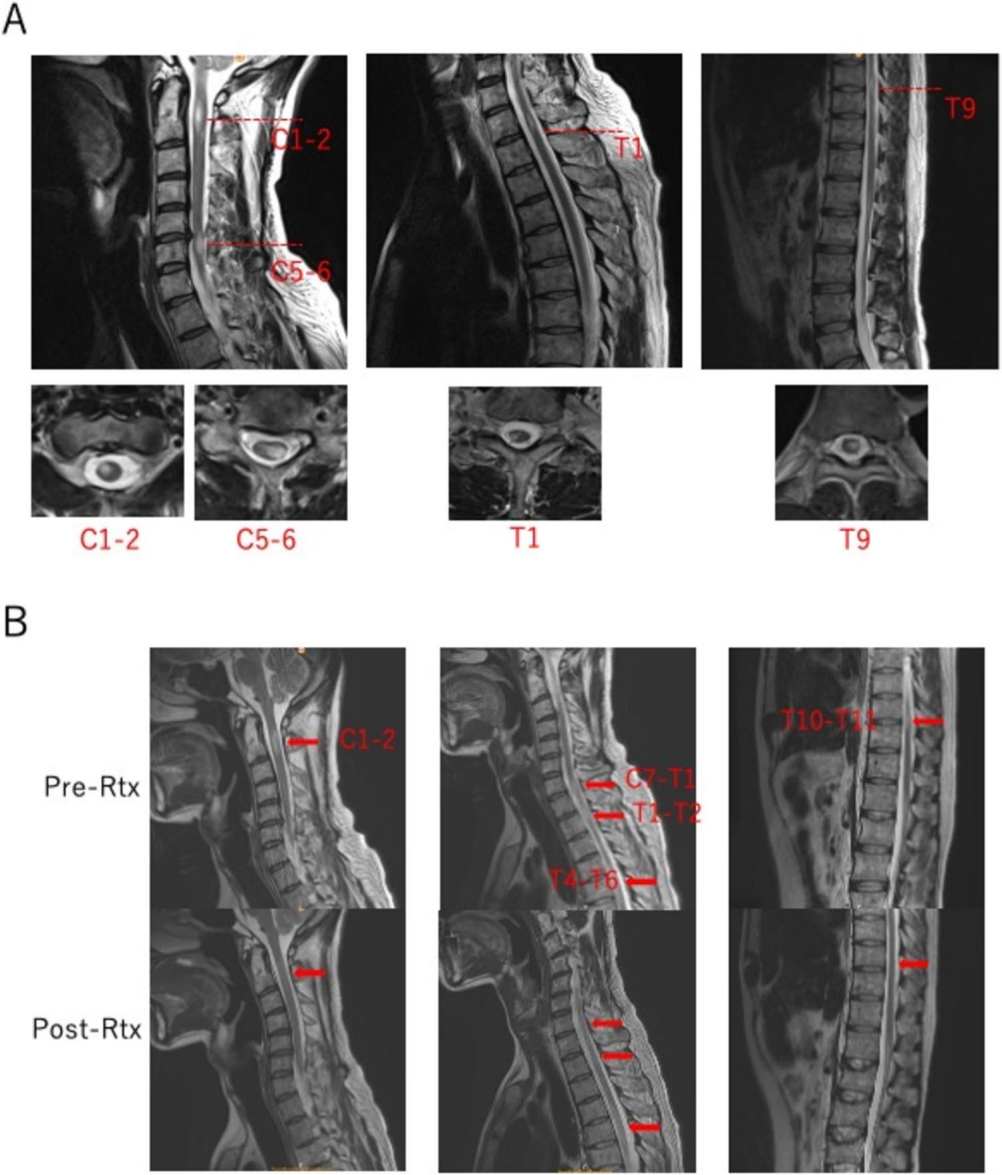
T2 weighted images of MRI **a** Abnormal lesions at the onset. High-intensity lesion observed at C1-2, C5-6, T1, and T9 levels. **b** Changes of abnormal signals at C1-C2, C7-T1, T1-T2, T4-T6, and T10-T11 levels pre and post rituximab treatment. Each abnormal signal of lesions attenuated after rituximab treatment

Lichenization of the oral cavity mucosa, score 2, moderate was diagnosed on day + 302. Soon after that, paralysis and paresthesia of the extremities and trunk predominantly on the left side suddenly occurred on day + 337 (Fig. 1). Magnetic resonance imaging (MRI) of the spinal cord showed multi-focal abnormal lesions. High-intensity lesions with T2WI at C1-2, C5-6, T1, and T9 levels of the spinal cord were observed (Fig. 2a). On the other hand, brain MRI showed no abnormality. Bladder and rectal disturbances also appeared, leading to urgent hospitalization. Cerebrospinal fluid (CSF) analysis revealed normal pressure and cell count (2/ul) and slightly elevated protein level (97 mg/dl). An oligoclonal band was not detected. IgG index was not elevated. Multiplex virus PCR of CSF showed no evidence of specific viral infection. MDS was in remission with complete donor chimerism. We diagnosed this condition as possible cGVHD of the CNS.

The dose of prednisolone was increased to 1 mg/kg from 4 days after admission, and intravenous immunoglobin (IVIG) was administered for 5 days at a dose of 400 mg/kg from 5 days after admission. However, the neurological symptoms rapidly deteriorated. Due to progressive bilateral paralysis of the lower extremities, the patient became completely unable to walk within a few days. We decided to switch to methyl-prednisolone pulse therapy (1000 mg/day, 3 days). After a pulse therapy, the scores of manual muscle tests (MMTs) of lower extremities improved from 0 to 4. However, after 13 days from pulse therapy, MMTs suddenly deteriorated to score 0. We started the second pulse therapy which resulted in improvement to score 4. Again, sudden deterioration occurred after 7 days from the second pulse therapy. We repeated one more pulse therapy, but slight improvement to score 2 was observed which persisted only 4 days. For the steroid-refractory clinical course, ibrutinib was initiated on day 23 of admission at a dose of 420 mg/day but was quickly discontinued due to the adverse effect of bleeding in the left-side elbow joint. Mycophenolate-mofetil (MMF) was also tested at a dose of 1000 mg/day from 1 month after admission and the dose was gradually increased to 3000 mg, but it showed no efficacy. Administration of IVIG was repeated monthly because it was partially effective for motor and sensory symptoms of the extremities. The dose of prednisolone was tapered after pulse therapy.

**Fig. 3:**
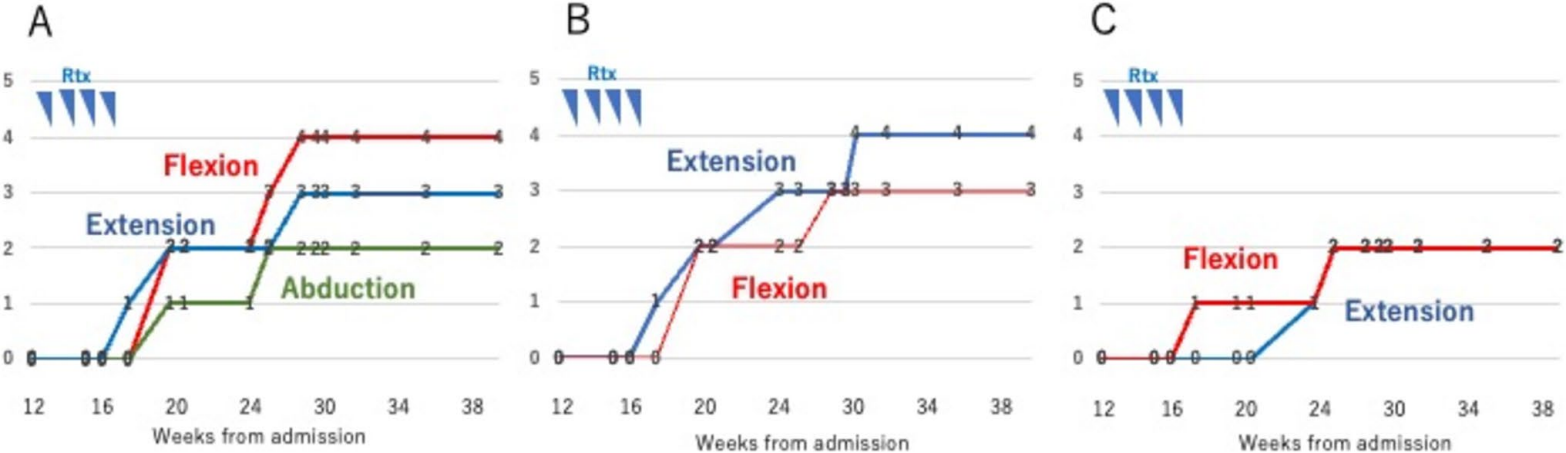
Transition of muscle manual tests (MMTs) of the lower extremities **a** Hip joint: From 0 to 4 for flexion, from 0 to 3 for extension, and from 0 to 2 for abduction **b** Knee joint: From 0 to 3 for flexion, and from 0 to 4 for extension **c** Ankle joint: From 0 to 2 for flexion, and from 0 to 2 for extension

After 3 months of hospitalization, he had left-side-dominant paralysis and paresthesia of the trunk below the T4 level. With bilateral paralysis and paresthesia of the lower extremities, he could not walk at all. The scores of all MMTs of the hip, knee, and ankle joints were 0 (Fig. 3a–c). There was also left arm paresthesia at C4–C8 levels. Abnormal lesions of spinal MRI were observed at C1-2, C7-T1, T1-T2, T4-6, T10-11 levels. Ibrutinib was a candidate of choice again, but we hesitated to use it considering the adverse effect of bleeding because the platelet count at that time was around 20–30 × 10^3^/μl. After approval of our institutional review board, rituximab at a dose of 375 mg/m^2^ was started at 14 weeks from the onset weekly for 4 times. Neurologic symptoms clearly improved after rituximab therapy, and MMTs of the lower extremities showed significant recovery (Fig. 3a–c). MRI of the spinal cord showed an attenuated T2WI high intensity signal of each lesion (Fig. 2b*)*. IVIG (400 mg/kg, 5 days) was continued monthly for 7 months from the onset followed by a maintenance dose of 500 mg/kg for 2 days in a 21-day cycle thereafter. With intensive rehabilitation, he regained the ability to walk with assistance, while the recovery of bladder and rectal disturbances was incomplete. He was discharged and continued treatment as an outpatient.

## Discussion

Diagnosis of cGVHD of the CNS is difficult without precise examination to rule out other complications of the CNS after allo-HSCT. However, treatment must be started as soon as possible from the onset because progression of symptoms can result in irreversible neurologic dysfunction. In our case, after confirming negativity of multiplex virus PCR of CSF, treatment with corticosteroid was started immediately. Repeated corticosteroid pulse therapy showed some efficacy, but it was temporary. IVIG was also started and repeated monthly, which showed slight efficacy each time. Because these treatments are commonly used for myelitis with various causes, we tried to continue them in the hope of amelioration. However, as a result, the patient’s lower extremities were almost completely paralyzed after 3 months.

For steroid-refractory cGVHD, various agents have been proposed and tested in preclinical and clinical investigations [7–9]. The development of cGVHD has complex and dynamic mechanisms and it is proposed that it has three biologic phases: early inflammation due to tissue injury, thymic injury and *T*-cell and *B*-cell dysregulation, tissue repair and fibrosis [10]. Although donor *T* cells clearly play a critical role in the initiation and maintenance of allo-immunity, many laboratory and clinical studies have shown that donor *B* cells are also important in the pathogenesis of cGVHD [11–14]. Ruxolitinib, ibrutinib, and belumosudil are recently approved drugs for steroid-refractory cGVHD treatment mainly targeting the second phase [15–17]. Axatilimab, which targets profibrotic macrophages and suppresses inflammation and fibrosis in the third phase, has also been introduced [18]. Rituximab has been historically one of proposed second-line treatments for cGVHD [19–21]. The overall response rate ranged from 40 to 80%, and it was effective especially for mild to moderate skin, oral cavity, and musculoskeletal manifestations. Rituximab did not seem to be effective for advanced cases with a severe NIH score. Therefore, the use of rituximab as salvage treatment for cGVHD has been restricted to specific conditions.

MS is an autoimmune disease that affects the CNS by demyelination. Although *T* cells have been recognized as the major contributors to the inflammatory activity in MS, growing evidence has shown the importance of the role of *B* cells [22, 23]. The effectiveness of the addition of *B*-cell-depleting therapies to current disease-modifying therapies for MS has been suggested by several studies [24, 25]. Rituximab has been demonstrated to reduce inflammatory activity, incidence of relapses and new brain lesions on MRI in patients with relapsing–remitting MS with low toxicity. It has also been shown that rituximab has an advantage in MS therapy with its relatively high blood–brain barrier penetration rate. Recently, ofatumumab, a fully human anti-CD20 monoclonal antibody, was approved for treatment of MS patients [26].

Our case had no abnormality in brain MRI, and an IgG oligoclonal band, which is one of the hallmarks of MS, was negative, but our case had some features shared with MS. There were differences between the multiple sites of abnormal lesions of the spinal cord at onset and those at 3 months after admission. It resembles the clinical course of MS in which the symptoms show progression and relief, disseminated in time and space. With these aspects, we decided to use rituximab among several treatment choices for steroid-refractory cGVHD, and rituximab was effective. Rituximab showed rapid and prolonged efficacy for the neurological symptoms, corresponding to attenuation of abnormal lesions of the spinal MRI. In the aspect of *B*-cell suppression and its well penetration rate to the CNS, ibrutinib might also have been effective in our case if it was not intolerant. In atypical cGVHD, there is no standard of care, especially in corticosteroid-refractory cases. Treatments that are used in corresponding autoimmune diseases may be reasonable and effective as shown in our case. Treatment with rituximab should be considered for cGVHD manifestations mimicking several autoimmune diseases like scleroderma, angiitis, and immune-mediated encephalitis or myelitis, in which rituximab has shown to be effective.

Treatment of cGVHD has been making progress in recent years with a better understanding of its mechanism and newly introduced drugs for steroid-refractory cases. The establishment of a treatment strategy for atypical cGVHD is also desirable in future. We should more aware the existence of atypical cGVHD and should use appropriate drugs earlier which is reasonable to the mechanism of each condition.

## Data Availability

The data that support the findings of this study are available on request from the corresponding author, E. Y. The data are not publicly available due to their containing information that could compromise the privacy of the reported subject.
